# Regulators of Cancer Progression: Succinylation

**DOI:** 10.3390/cancers17162652

**Published:** 2025-08-14

**Authors:** Jie Gao, Wei Yu

**Affiliations:** 1 Institute of Biochemistry, College of Life Sciences and Medicine, Zhejiang Sci-Tech University, Hangzhou 310018, China; 2024220903004@mails.zstu.edu.cn; 2 Zhejiang Provincial Key Laboratory of Silkworm Bioreactor and Biomedicine, Hangzhou 310018, China

**Keywords:** succinylation, cancer, tumor, drug

## Abstract

Lysine succinylation is a recently discovered post-translational protein modification, the process of which requires the participation of various enzymes. The close association between cancer and protein post-translational modifications (PTMs), such as acetylation and phosphorylation, has been extensively investigated and well-established. In recent years, growing attention has been directed toward the role of succinylation in cancer progression. Accumulating evidence demonstrates that protein succinylation and desuccinylation play critical roles in promoting the development of various cancers, including lung, prostate, and renal cancers. Notably, the primary substrates undergoing succinylation are non-histone proteins. Therefore, elucidating the functions of cancer-related succinylated proteins is essential for identifying novel therapeutic targets. This review comprehensively summarizes current research advances regarding protein succinylation in common cancers and discusses the progress in developing succinylation-targeting drugs. Specifically, we focus on the molecular mechanisms by which succinylation regulates cancer progression, along with the identification of key succinylation sites. Our discussion aims to provide valuable insights for future research and the development of innovative cancer treatments.

## Simple Summary

Succinylation, as a newly discovered form of protein post-translational modification (PTM), lacks systematic characterization regarding its relationship with cancer. This review investigates the mechanisms of protein succinylation in nine common cancer types as well as other malignancies, highlighting its potential as a promising therapeutic target. Furthermore, we summarize recent advances in drug development targeting succinylation. Finally, we discuss the current limitations of this research field and provide perspectives for future studies on succinylation in cancer.

## 1. Introduction

Post-translational modifications (PTMs) are key processes that control how proteins work, where they are located, and how they interact with other molecules. They greatly affect important biological activities like cell signaling, metabolism, and the passing on of genetic information [1]. Lysine succinylation, a novel PTM, was first discovered in *E. coli* by Zhang et al. in 2011 [2]. It dynamically regulates the activity and function of proteins by covalently attaching succinyl groups to lysine residues of substrate proteins. Succinylation has since been discovered in other bacteria as well as mammals [3]. Compared to other acylation modifications, succinylation is more specific to the regulation of protein structure and function due to its larger moiety with a negative charge [4].

Succinyl coenzyme A (succinyl-CoA) is the main source of succinyl groups. Its levels are carefully controlled by several metabolic processes. These include the tricarboxylic acid (TCA) cycle, the breakdown of branched-chain amino acids, and the beta-oxidation of odd-chain fatty acids. This regulation ensures a steady supply of succinyl groups. In addition to succinyl-CoA, the vast majority of succinylation reactions in cells require the action of succinyltransferases and desuccinylases. With the coordination of these two enzymes with opposite functions, various histones or non-histone proteins are succinylated and desuccinylated to maintain physiological homeostasis.

Recent studies have shown that succinylation is not only widely involved in basic biological processes such as cellular energy metabolism, chromatin remodeling, and DNA damage repair, but is also closely related to the development of cancer [5,6,7]. For example, succinimidylation modifications are involved in the development and malignant progression of many cancers by specifically affecting chromatin structure and gene transcription by modifying key lysine residues of histones [8]. Studying these special modification sites in detail will help find better molecular targets and drug strategies. This can lead to early cancer diagnosis, better prevention, and precise treatment. This research direction has important translational value and clinical application prospects.

Cancer is a complex class of diseases caused by the uncontrolled proliferation and spread of abnormal cells, which has become the second leading cause of death worldwide [9,10]. When the body’s own cells accumulate mutations due to genetic or environmental factors, it eventually leads to the failure of the regulatory mechanisms of cell proliferation, differentiation, or apoptosis. As a consequence, cancer cells evade the fate of normal cells, and eventually break through tissue boundaries to invade surrounding structures. Finally, cancer spreads to distant organs through the blood or lymphatic system [11]. While most cancers are solid tumors, such as lung adenocarcinoma and glioblastoma [12], a few are hematological malignancies, such as lymphoma [13].

This article describes the regulatory mechanism of succinylation and its association with various common cancers, with a view to informing potential targets for pharmaceutical treatment.

## 2. Lysine Succinylation

### 2.1. Succinyl-CoA

**Figure 1:**
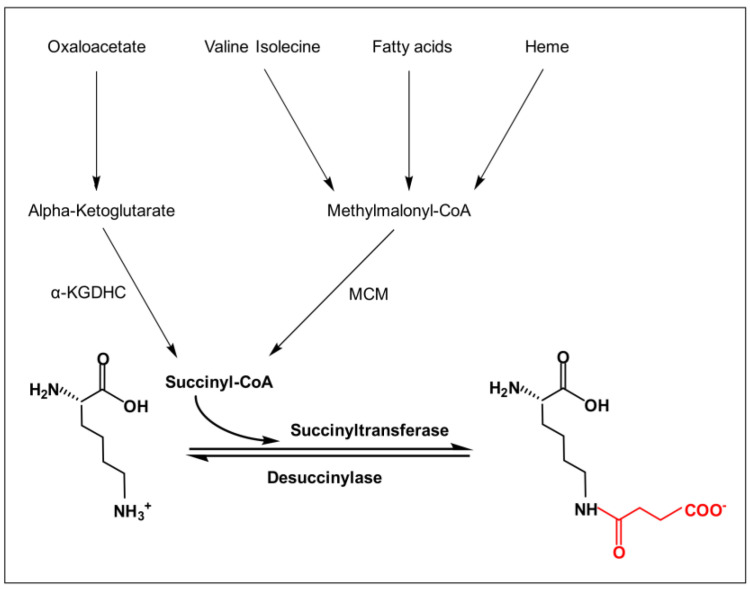
Lysine succinylation process. The figure illustrates the process of lysine undergoing succinylation modification in the presence of succinyl-CoA, succinyl transferase, and desuccinylase. Conversion of alpha-ketoglutarate from oxaloacetic acid leads to the formation of succinyl-CoA via α-KDGHC. Valine, isoleucine, and heme lead to methylmalonyl CoA, which is then catalyzed by the MCM to produce succinyl-CoA. The charge of the lysine side chain changes before and after the reaction, transforming from a positively charged −NH_3_^+^ to a negatively charged −COO^−^.

Non-enzymatic succinylation is mainly regulated by changes in the levels of succinyl-CoA, the major succinyl donor [5]. The succinyl-CoA production process can be divided into two parts (Figure 1). The main source of succinyl-CoA is the TCA cycle [14], as the oxidative decarboxylation of α-ketoglutarate catalyzed by the alpha-ketoglutarate dehydrogenase complex (α-KGDHC) generates succinyl-CoA [15]. Earlier, it was also found that the catabolism of valine, isoleucine, and leucine also generates succinyl-CoA as part of branched-chain amino acid metabolism. The β-oxidation of odd-chain fatty acids produces propionyl coenzyme A, which is converted into succinyl-CoA by propionyl coenzyme A carboxylase and methylmalonyl coenzyme A mutase (MCM) [16]. In addition, the catabolism of heme to biliverdin, catalyzed by heme oxygenase (HO), also releases succinyl-CoA [17].

### 2.2. Enzymatic Succinylation

Enzymatic succinylation tends to be more prevalent and important in organisms because of its high efficiency and specificity compared to non-enzymatic succinylation. The process generally involves specific succinyltransferases and desuccinylases (Figure 1; Table 1). Succinyltransferases are enzymes that covalently attach succinyl groups to lysine residues. Succinyltransferases have been identified in two broad acyltransferase families, including the histone acetyltransferase (HAT) family and the carnitine palmitoyltransferase (CPT) family. The HAT family of succinyltransferases includes P300/CBP [18], lysine acetyltransferase 2A (KAT2A) [19], and histone acetyltransferase 1 (HAT1) [20]. P300 and CBP are two closely related transcriptional co-activator proteins, which are structurally and highly functionally similar, and therefore collectively referred to as P300/CBP [21]. The CPT family is dominated by carnitine palmitoyltransferase 1A (CPT1A) [22]. Desuccinylases catalyze the opposite reaction to succinyltransferases, with the two main representatives being sirtuin 5 (SIRT5) [23] and sirtuin 7 (SIRT7) [24]. They are all part of the sirtuin family, also called class III histone deacetylases (HDACs), which need nicotinamide adenine dinucleotide (NAD^+^). This allows different histones and non-histone proteins to be changed by adding or removing succinyl groups, with the help of succinyl donors, succinyltransferases, and desuccinylases.

## 3. Roles of Succinylation in Different Cancers

**Figure 2:**
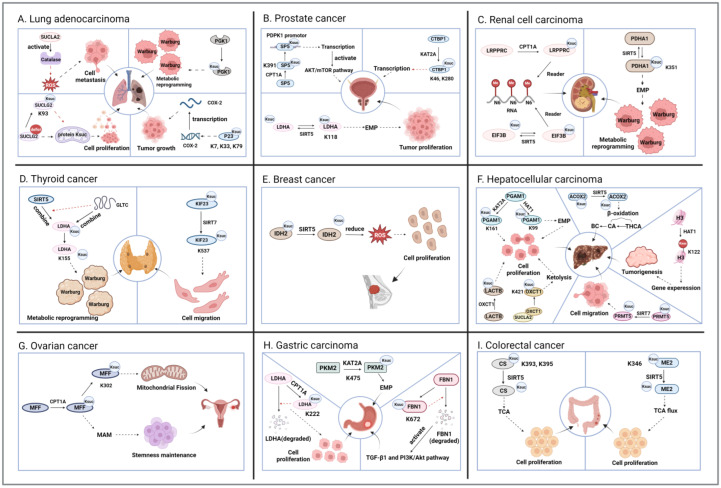
The mechanism of action of succinylation in cancer. The figure shows the succinylation modification in nine common types of cancer. Both black dotted lines and gradient lines represent promotion, and red dotted lines represent inhibition. (**A**). Lung adenocarcinoma: SUCLA2 and SUCLG2 influence the succinylation of related proteins through their concentrations, thereby promoting cell migration and proliferation; PGK1 and P23 self-succinylation, respectively, promote tumor metabolic reprogramming and tumor growth. (**B**). Prostate cancer: The succinylation of SP5, CTBP1, and LDHA proteins affects transcription and glycolysis. (**C**). Renal cell carcinoma: The succinylation of LRPPRC and EIF3B proteins affects RNA modification, while PDHA1 succinylation promotes metabolic reprogramming. (**D**). Thyroid cancer: Desamidation of LDHA and KIF23 promotes metabolic reprogramming and cell migration. (**E**). Breast cancer: Desamidation of IDH2 promotes cell proliferation. (**F**). Hepatocellular carcinoma: Self-succinylation of PGAM1, LACTB, and OXCT1 promotes cell proliferation; desuccinylation of ACOX2 aids in BC generation; succinylation of H3 promotes tumorigenesis; desuccinylation of PRMT5 increases cell migration. (**G**). Ovarian cancer: Succinylation of MFF promotes mitochondrial fission and maintenance of stem cell pluripotency. (**H**). Gastric cancer: PKM2 succinylation promotes EMP, while LDHA and FBN1 succinylation inhibit self-degradation. (**I**). Colorectal cancer: Both CS and ME2 desuccinylation promote cell proliferation via the TCA cycle. Figure created using BioRender.com (accessed on 13 August 2025).

**Figure 3:**
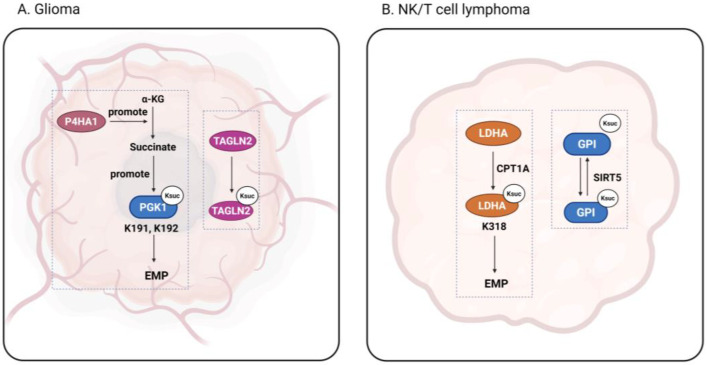
The function of succinylation in glioma and lymphoma. The figure shows succinylation occurring in glioma and nasal-type extranodal natural killer/T-cell lymphoma. Figure created using BioRender.com (accessed on 9 August 2025).

Cancer, one of the leading causes of death worldwide, continues to have an increasing incidence and mortality rate [45]. Early studies have discovered the upregulation and downregulation of specific succinylation sites in colorectal cancer cells using proteomics techniques. Subsequent studies have found significant differences in the succinylation levels of various proteins through different metabolic pathways in gastric cancer [46]. Emerging research has further demonstrated that succinylation can regulate cancer progression by modifying key enzymes in metabolic pathways, such as the TCA cycle, which is an important supplement to the cancer formation mechanism. In addition to metabolic proteins, the succinylation of many other proteins, including histones and non-histones, plays a significant role in cancer progression. The addition or removal of succinyl-lysine (Ksuc) on proteins with different functions jointly promotes the proliferation and migration of cancer cells or the formation of tumors, ultimately leading to cancer. This article summarizes the regulatory mechanisms of nine types of cancer related to succinylation and two types of malignant tumors (Figure 2 and Figure 3).

### 3.1. Lung Adenocarcinoma

Lung cancer is the second most common type of cancer and the leading cause of cancer-associated mortality worldwide [47]. Lung adenocarcinoma (LUAD) is one of the most common subtypes, accounting for approximately 40% of all lung cancer cases [48,49]. Succinyl-CoA synthetase GDP-forming subunit β (SUCLG2) is an important component of succinyl-CoA synthetase (SUCL) [50]. It has been shown that deletion of SUCLG2 can increase the succinylation level of mitochondrial proteins and thus inhibit mitochondrial function in LUAD cells. When succinyl-CoA synthetase activity decreases, it stops making succinyl-CoA. This causes succinate to build up and succinyl-CoA to form through a non-enzymatic process, which then increases protein succinylation. However, SUCLG2 itself is concurrently succinylated at Lys93, which increases its protein stability, decreases the succinylation of mitochondrial proteins, and promotes the proliferation of LUAD cells [51]. Hence, there is a feedback loop mediated by SUCLG2. In addition, as another β-subunit of SUCL, the succinyl-CoA synthase ADP-forming subunit β (SUCLA2) has also been shown to be closely associated with various cancers [52,53]. Through mitochondrial RNAi screening, SUCLA2 was identified as a key loss-of-nest apoptosis resistance and metastasis driver in human cancers. In the cytoplasm, SUCLA2 can attach to and turn on antioxidant proteins like catalase. It also helps form stress granules. These actions lower the levels of reactive oxygen species (ROS) inside cells and make cancer cells more resistant to apoptosis when they lose their attachment to a surface [54]. P23 is often considered a co-chaperone of heat shock protein 90 (HSP90) [55], but its biological function independent of HSP90 is also gradually being explored. Yu et al. found that p23 is a previously uncharacterized transcription factor of the cyclooxygenase 2 (COX-2) gene. Abnormal accumulation of succinate in the LUAD tumor environment promotes succinylation of p23 at K7, K33, and K79, which drives their nuclear translocation for COX-2 transcription, thereby inducing tumor growth. When p23 succinylation and nuclear translocation are inhibited by M16, tumor growth is also inhibited [56]. This shows that p23 might become a new marker for cancer by targeting succinylation [57]. Ma et al. found that p300 adds a succinyl group to certain proteins, including phosphoglycerate kinase 1 (PGK1-K323), phosphoenolpyruvate carboxylase 2 (PCK2-K108), phosphofructokinase 1 (PFKL-K677), pyruvate kinase (PKM-K166), and aldolase A (ALDOA-K13). This action changes how cells process glucose in LUAD. Among these, PGK1 is most affected by the succinylation from p300 [25].

### 3.2. Prostate Cancer

After lung cancer, prostate cancer (PCa) is the second most common malignancy in men [58]. A study of succinylation in PCa showed that tumor tissue had significantly higher levels of succinyl-lysine than healthy paraneoplastic tissues, and which was correlated with the stage of PCa [59]. C-terminal binding protein 1 (CTBP1) has been shown to be a co-repressor of downstream gene transcription, and is involved in a variety of cellular processes [60]. Recent studies have found that CTBP1 is highly expressed in PCa tissues and cell lines, where it exerts a promoting effect on cancer progression. CTBP1 can bind to SP1 and inhibit CDH1 transcription, while KAT2A-mediated succinylation of CTBP1 at K46 and K280 upregulates the transcriptional repressor activity of CTBP1, thus promoting PCa progression [28]. Aberrant activation of the AKT/mTOR signaling pathway is often closely associated with cancer development [61]. Phosphoinositide-dependent protein kinase-1 (PDPK1) is a core kinase in the PI3K-Akt-mTOR signaling pathway. CPT1A helps to add a succinyl group to specificity protein 5 (SP5) at position K391. This modified SP5 then attaches to the PDPK1 promoter, which turns on the PDPK1 gene. This activation leads to the AKT/mTOR signaling pathway being turned on in PCa cells [32], thus promoting cancer progression. Lactate dehydrogenase A (LDHA) is an isoenzyme of lactate dehydrogenase that is mainly responsible for regulating the final step of glycolysis. Previous studies have shown that the expression level of LDHA is significantly higher in PCa than in normal tissues, especially in invasive and metastatic tumors. Recent studies found that increased succinylation of LDHA at K118 promotes LDHA activity and facilitates glycolysis [36]. This finding provides a new molecular mechanism for understanding PCa progression and suggests that LDHA succinylation may be a potential target for PCa therapy.

### 3.3. Renal Cell Carcinoma

Renal cell carcinoma (RCC) is a strikingly heterogeneous malignant tumor originating from renal tubular epithelial cells. Patients with RCC often have other systemic diseases, which affect treatment options and prognosis [62,63]. RCC mainly includes three subtypes, one of which is clear cell RCC (ccRCC). Lu et al. [33] found that CPT1A, KAT2A, SIRT5, and SIRT7 play critical roles in the prognosis of ccRCC, and specifically promote its malignant progression by regulating the infiltration of immune cells and the methylation of RNA N6-methyladenosine (m6A). The latter modification is regulated by factors such as HNRHPA2B1, HNRNPC, RBMX, EIF3B, and LRPPRC to promote cancer development [64]. In ccRCC cells, CPT1A and SIRT5 can, respectively, up- and downregulate the expression of LRPPRC and EIF3B, which are both m6A “readers” [65,66]. This means CPT1A might help keep LRPPRC stable through succinylation. On the other hand, SIRT5 might make EIF3B less stable through desuccinylation. More experiments are needed to confirm this. Another study found that SIRT5 knockdown promoted ccRCC tumorigenesis and metastasis. When SIRT5 expression is reduced in ccRCC, it causes hypersuccinylation at the K351 site of pyruvate dehydrogenase α1 (PDHA1) [37]. This lowers the activity of the pyruvate dehydrogenase complex (PDC), which then increases glycolysis in tumor cells. This contributes to the Warburg effect [67]. This metabolic reprogramming process ultimately promotes the malignant transformation and metastatic spread of ccRCC, providing a new perspective for understanding the pathogenesis of this malignancy [37]. Fumarate hydratase (FH)-deficient RCC is a unique subtype whose genome has recently been characterized [68]. FH is an enzyme in the TCA cycle responsible for converting fumarate into malate. It is well-established that the loss of FH disrupts TCA cycle activity, resulting in the accumulation of fumarate and triggering metabolic reprogramming in tumors [69]. Succinylcysteine (Csucc) and succinyladenosine (Ado-succ) are two circulating metabolites identified by high-sensitivity MS-based metabolic profiling that reliably reflect FH mutant status and tumor mass [70]. Thus, they may serve as sensitive and specific biomarkers for early diagnosis and prognosis of FH-deficient RCC.

### 3.4. Thyroid Cancer

Thyroid cancer (TC) is a common endocrine malignancy with an optimistic 5-year relative survival rate of 98.5%, albeit with an increasing incidence [71]. Papillary thyroid cancer (PTC) is the most common histological type of TC. Similarly to one of the mechanisms in prostate cancer, PTC progression can also be promoted by the succinylation of LDHA through SIRT5 downregulation. The long-chain non-coding RNA (lncRNA) GLTC can competitively inhibit the interaction between SIRT5 and LDHA and thus promote the succinylation of LDHA on K155, reducing SIRT5 levels [35]. LncRNAs have been shown to be aberrantly expressed in various cancers, and have the potential to serve as a biologic therapeutic target [72]. Undifferentiated thyroid carcinoma (also known as anaplastic thyroid cancer, ATC) is a particularly malignant subtype of TC [73]. Kinesin family member 23 (KIF23) is a kinesin family member that is widely involved in mitosis, and its upregulation is often associated with tumor development [74]. Recently, it was found that upregulation of KIF23 expression in ATC cells promotes their migration. Further studies revealed that SIRT7 can maintain the protein stability of KIF23 and its pro-tumorigenic activity by specifically inhibiting its succinylation at K537 [43]. This SIRT7-mediated desuccinylation was found to not only enhance the biological function of KIF23, but also significantly promote the proliferation and migration of ATC cells, providing a new molecular perspective for understanding the invasive metastatic behavior of this aggressive malignancy.

### 3.5. Breast Cancer

Breast cancer (BC) is one of the major diseases endangering women’s health, and its incidence has been increasing in recent years [75]. Liu et al. detected for the first time the regulation of lysine succinylation in BC progression. Succinylation of pentose phosphate pathway (PPP)-related metabolic enzymes was upregulated in BC cells compared to normal tissues, with the most significant changes in transketolase (TK) [76]. As a histone protein, H2A.X mainly plays a role in the response to cellular DNA damage [77]. However, a recent proteomic study revealed that the succinylation levels of most proteins were significantly higher in BC tissues than in normal tissues, and the modified proteins were significantly enriched in three H2A.X complexes [78]. Among them, nuclear phosphoprotein (NPM1) was simultaneously acetylated and succinylated at K27, a site highly conserved in several species, which may contribute to BC progression by modulating the chromosomal structure to influence the DNA damage response [79]. Nevertheless, the exact mechanism requires further investigation. Normal cells do not require SIRT5 for survival, but one study found that BC cells are addicted to SIRT5 for anchorage-independent growth [38]. Anchorage-independent growth leads to an increase in reactive oxygen species (ROS), and the presence of SIRT5 leads to the desuccinylation of isocitrate dehydrogenase (IDH2), a key enzyme in the antioxidant response, which attenuates ROS and promotes cancer cell growth. As a consequence, SIRT5 inhibitors can attenuate the transforming properties of cultured BC cells and significantly reduce breast tumor growth in vivo.

### 3.6. Hepatocellular Carcinoma

Hepatocellular carcinoma (HCC) is the sixth most common malignant tumor worldwide, and its occurrence is often accompanied by viral infections and liver cirrhosis [80,81]. Yang et al. reported that HAT1 expression was significantly upregulated in HCC. Further studies revealed that HAT1 catalyzes the succinylation of histone H3 at K122, which enhances gene expression in tumor cells through epigenetic regulation [20]. In addition, HAT1 can promote glycolysis by catalyzing the succinylation of phosphoglycerate mutase 1 (PGAM1) on K99 in cancer cells, thereby promoting tumorigenesis. PGAM1 catalyzes the conversion of 3-phosphoglycerate (3-PG) to 2-phosphoglycerate (2-PG) in glycolysis; thus, its succinylation can promote glycolysis. In addition, Zhu et al. [26] found that KAT2A can also use PGAM1 as a succinylation substrate. KAT2A enhances the viability and glycolysis of HCC cell lines by regulating the succinylation of PGAM1 at K161, leading to tumor progression. Unlike the molecular target of arginine methyltransferase 1 (PRMT1) in ccRCC, arginine methyltransferase 5 (PRMT5) plays an important role in HCC cells [82]. It is not only involved in lipid metabolism reprogramming, but also significantly promotes tumor growth and metastasis. The specific mechanism depends on SIRT7-mediated desuccinylation of PRMT5 at K387 in tumors [44]. SIRT7-mediated desuccinylation of PRMT5 K387 promotes HCC cell proliferation, migration, and invasion in vitro, while also accelerating tumor growth and metastasis in vivo. Sun et al. [39] found that the absence of SIRT5 promoted the increase in bile acid (BC) synthesis and thus induced hepatocarcinogenesis. This is partly due to the fact that reduced SIRT5 expression can lead to increased lysine succinylation of branched-chain acyl-coenzyme A oxidase 2 (ACOX2). ACOX2 is responsible for catalyzing the β-oxidation of the primary bile acid precursor, taurohyocholic acid, into the primary bile acid (cholic acid, CA), and subsequently into BC. β-lactamase-like protein (LACTB) is a β-lactamase-like protein located mainly in mitochondria and has been found to function as a tumor suppressor [83,84]. In HCC cells, OXCT1 was recently found to promote the succinylation of LACTB at K284, which inhibits its protein hydrolysis activity, thus promoting the proliferation of tumor cells and ultimately leading to the progression of HCC [34]. While previous studies demonstrated that OXCT1 can function as a succinyltransferase, recent studies found that it can also act as a rate-limiting enzyme for ketolysis in HCC cells. In this context, insulin-like growth factor 1 (IGF1) stimulates OXCT1 to interact with SUCLA2, resulting in the K421 succinylation and subsequent activation of OXCT1. This occurs due to the SUCLA2-catalyzed production of succinyl-CoA, which promotes ketolysis and the proliferation of HCC cells [85]. In addition, Wang et al. found a relatively significant crosstalk between propionylation and succinylation in a comparative study of PTMs between tumor tissues and normal tissues of HCC patients, suggesting that this pair of PTMs may be related to many protein functions [86]. However, their exact roles in HCC progression need further study.

### 3.7. Ovarian Cancer

Ovarian cancer (OC) is one of the most severe female-specific malignancies, with approximately 140,000 women worldwide dying from OC each year [87]. Mitochondrial fission factor (MFF), an important regulator of mitochondrial fission and function, tends to be highly expressed in cancer cells, and dysregulated mitochondrial fission has oncogenic effects [88,89]. Recent studies found that succinylation of MFF affects OC progression. CPT1A succinylates MFF at K302, which can prevent its parkin-mediated ubiquitin-dependent proteasomal degradation, thus stabilizing the expression of MFF [29]. Enhanced activity of MFF in turn promotes mitochondrial fission and facilitates the development of OC. In addition, MFF has a promoting effect on the formation of mitochondria-associated membranes (MAMs). Proteins involved in MAM formation have been implicated in tumorigenesis and cancer stem cell maintenance [90]. CPT1A-mediated MFF succinylation promotes the formation of MAMs, which supports the maintenance of OC stemness and can contribute to the progression of OC as well as increase drug resistance [91]. Therefore, MFF succinylation represents a potential therapeutic target.

### 3.8. Gastric Cancer

Gastric cancer (GC), a major cancer of the gastrointestinal tract, is the fifth most common malignant tumor worldwide [92,93]. In recent years, the link between GC and succinylation is also increasingly being explored. Li et al. [30] found that LDHA was significantly upregulated in GC cells compared to normal cells. Subsequent research revealed that LDHA undergoes modification at the K222 site, which hinders its ability to bind with sequestosome 1 (SQSTM1). Consequently, LDHA becomes less susceptible to degradation in lysosomes. This succinylation process is regulated by CPT1A, while high succinylation of LDHA K222 promotes GC cell invasion and proliferation. The extracellular matrix (ECM) is essential for tumorigenesis as well as cancer progression, and extracellular matrix protein fibrilin-1 (FBN1) is a major component of the ECM [94]. Similarly to LDHA, succinylation of FBN1 at the K672 site blocks its degradation by matrix metalloproteinases (MMPs). Long-term accumulation and deposition of FBN1 promotes GC tumor progression through activation of TGF-β1 and the intracellular PI3K/Akt pathway [95]. It is therefore evident that succinylation of both LDHA and FBN1 is favorable for cancer development. Pyruvate kinase M2 (PKM2) is a rate-limiting glycolytic enzyme whose low activity is required for glycolysis in cancer cells [96]. KAT2A succinylates PKM2 at K475, which inhibits its activity rather than altering its protein levels, thus allowing GC cells to enhance glycolytic metabolism and ultimately promoting cancer progression [27].

### 3.9. Colorectal Cancer

Colorectal cancer (CRC) is the third most common malignancy in the world, with a progressively younger incidence group [97]. The role of succinylation in CRC has also become increasingly apparent in recent years. Citrate synthase (CS), the rate-limiting enzyme catalyzing the first step of the TCA cycle, condenses oxaloacetate with acetyl coenzyme A to produce citrate [98]. In CRC cells, the two highly conserved lysines 393 and 395 are hypersuccinylated, which reduces the enzymatic activity of CS to inhibit CRC cell proliferation and migration [40]. However, SIRT5 is able to reverse this succinylation to promote cancer progression. Mitochondrial malic enzyme 2 (ME2) is an important regulator of TCA flux [99], and it was found that succinylated ME2 is highly abundant in CRC cells. Moreover, the succinyl modification of its K346 site can also be removed by SIRT5, leading to an enhancement of ME2 enzyme activity, which in turn promotes mitochondrial respiration and cancer cell proliferation [41]. It is therefore evident that SIRT5-mediated desuccinylation can promote CRC progression.

### 3.10. Gliomas and Lymphomas

Gliomas, which can be both benign and malignant, are the most common primary brain tumors in adults and children [100]. Transgelin-2 (TAGLN2), a member of the transgelin family of actin-binding proteins, has been found to play an important role in tumor progression and malignant transformation [101]. Zhang et al. found that the TAGLN2 K40 site is highly succinylated in glioma endothelial cells (GECs), contributing to angiogenesis and tumor growth [102]. Thus, inhibiting the succinylation of TAGLN2 may be a promising strategy for the treatment of gliomas. Glioblastoma (GBM) is among the most malignant gliomas, accounting for approximately 49% of all malignant brain tumors [103]. Yang et al. team found that in GBM, proline 4-hydroxylase α-subunit (P4HA1) activates the HIF1α pathway by increasing the cytoplasmic concentration of succinic acid, which increases the overall succinylation level of the protein. At the same time, PGK1, a key glycolytic enzyme, is subjected to high succinylation at the K191 and K192 sites, which in turn enhances glycolysis in tumor cells and aids tumor growth [104].

Nasal-type extranodal natural killer/T-cell lymphoma (ENKTL-NT) is a subtype of non-Hodgkin’s lymphoma that is derived from transformed natural killer cells and is usually found in the nasal cavity [105]. Lymphomas are often accompanied by T-cell defects, affecting cancer immunotherapy [106]. Wu et al. [31] revealed that glycolysis is the main metabolic mode of ENKTL-NT, whereby succinylation of LDHA at the K318 site helps to improve its stability and further promote glycolysis. This succinylation is mediated by CPT1A, in congruence with the upregulation of CPT1A in ENKTL-NT tissues. Wang et al. [107] investigated another key enzyme of glycolysis, glucose-6-phosphate isomerase (GPI), which catalyzes the second step of glycolysis. In ENKTL-NT cells, GPI undergoes succinylation, but its succinylation is inhibited in the presence of SIRT5, and tumor growth is blocked. It is thus clear that succinylation of glycolytic enzymes is inextricably linked to ENKTL-NT tumor development, which has important reference value for future tumor drug targeting.

## 4. Potential Anticancer Drugs Targeting Succinylation

Since cancer has invaded the world, the research and development of anticancer drugs has always been an important strategy for fighting cancer. With the help of technologies like artificial intelligence, many identified compounds have been shown to effectively slow down cancer growth [108,109]. The connection between these compounds and succinylation has also been found in some studies in recent years, but they have not yet been put into clinical trials. The research status of the targeted succinylated compounds is shown in Table 2. Aspirin is a non-steroidal anti-inflammatory drug, which has been shown to have certain effects on cancer [42]. NF-κB is an important inflammation-related factor in the body [110]. Recent studies show that aspirin can stop PGAM1 succinylation at K99. It does this by targeting NF-κB p65 to reduce HAT1 in liver cancer tissues. This limits PGAM1 activity and glycolysis [111]. Moreover, astragaloside IV (AS-IV), a natural compound derived from Chinese herbal medicine, also inhibits KAT2A-mediated succinylation of PGAM1 and consequently glycolysis, demonstrating effectiveness in HCC cells. This suggests that AS-IV could be a promising and appropriate therapeutic agent for treating HCC [26]. Lidocaine is a widely used amide-based local anesthetic that has been shown to inhibit the progression of many cancers. HIST1H2BL is a gene that is abnormally expressed in lung cancer, and its protein has succinylation sites [112]. Chen et al. [113] found that lidocaine can inhibit the progression of LUAD by promoting the SIRT5-mediated desuccinylation of HIST1H2BL. Glibenclamide is a sulfonylurea compound that is primarily used to treat diabetes [114]. Recent studies have revealed that glibenclamide can inhibit the lysine succinyltransferase activity of CPT1A, thereby reducing MFF succinylation. This, in turn, leads to a decrease in the stemness of ovarian cancer stem cells (OCSCs), offering a potential target and therapeutic strategy for OC treatment [91]. In addition, PCa cells harboring an RB1-SUCLA2 deletion are metabolically unstable and sensitive to thymoquinone (2-isopropyl-5-methylbenzo-1,4-quinone) as well as PMA (phorbol-12-myristate-13-acetate) [115]. Catalpol is an iridoid glycoside derived from the traditional Chinese medicinal plant Rehmannia glutinosa. Similarly to natural compounds such as berberine, it can induce apoptosis in breast cancer cells [116]. Liu et al. [117] found that succinylation in BC tumors was significantly reduced by catalpol treatment, hinting at the possibility of clinical translation. In addition, histone succinylation can also affect the resistance of cancer cells to chemotherapeutic agents. In pancreatic cancer cells, knockdown of SIRT7 resulted in increased expression of glucose transporter protein 3 (GLUT3) as well as increased levels of H3K122succ, which in turn led to pancreatic cancer cells being more sensitive to gemcitabine.

Zhang et al. [118] discovered that the differentially expressed gene KLF6 could recruit P300, leading to elevated levels of H3K23succ in colorectal cancer cells, which subsequently became more resistant to 5-fluorouracil [119,120]. This provides a reference for addressing the problem of drug resistance in targeted cancer therapy.

## 5. Conclusions

The mechanism of action and pharmacological targeting of novel PTMs in cancer have become a focus of research. This paper reviews recent advances in our understanding of the roles played by protein lysine succinylation in promoting or inhibiting cancer, as well as recent advances in drugs targeting succinylation. Succinylation is prevalent in cells, affecting histones and transcription factors within the nucleus, as well as non-histone proteins like metabolic enzymes in the cytoplasm. These proteins undergo succinylation or desuccinylation during cancer development, thereby indirectly influencing tumor progression. Therefore, these succinylated substrates can be further investigated as drug targets for clinical treatment in the future. In addition, inhibitors and activators can be developed for related enzymes to better intervene in the succinylation process. A modulator of SIRT5 and SIRT7 has been developed to intervene in cancer, with promising results [121].

While numerous studies have focused on succinylated proteins in cancer, these proteins represent just a small fraction of the myriad mechanisms involved in cancer. Their roles in overall signaling pathways and their interactions with other post-translational modifications (PTMs) remain largely unexplored. In addition, to date, the specific targets of action of succinylation modifications and their functional heterogeneity in different types of malignancies still lack systematic studies. The mechanism of selective recognition of substrates by succinyltransferases and desuccinylases, as well as the complex regulatory network they participate in, need to be comprehensively analyzed using multi-omics approaches. At the same time, the clinical applicability, safety, and efficacy of therapeutic strategies developed for succinylation modifications still need to be verified and improved through a large number of basic studies and clinical trials. The resolution of these key scientific issues will facilitate the transition of succinylation-targeting therapies from laboratory research to clinical application.

## Figures and Tables

**Table 1:** Regulatory enzymes of succinylation, their classification, and years of discovery.

Regulation Enzyme	Family	Members	Succinylation Target Proteins	Year	Refs.
	HAT	P300/CBP	PGK1	2024	[25]
		KAT2A	PGAM1 (K161), PKM2 (K475), CTBP1 (K46, K280)	2024, 2024, 2023	[26,27,28]
Succinyltransferases		HAT1	H3 (K122), PGAM1 (K99)	2021	[20]
	CPT	CPT1A	MFF, LDHA (K222), LDHA (K318), SP5 (K391), LRPPRC	2023, 2020, 2025, 2024, 2021	[29,30,31,32,33]
	CoAT	OXCT1	LACTB	2024	[34]
Desuccinylase	Sirtuin	SIRT5	LDHA (K155), LDHA (K118), EIF3B, PDHA1 (K351), IDH2, ACOX2, CS (K393, K395), ME2 (K346), GPI	2023, 2023, 2021, 2021, 2021, 2022, 2020, 2024, 2025	[33,35,36,37,38,39,40,41,42]
		SIRT7	KIF23 (K537), PRMT5	2024, 2022	[43,44]

**Table 2:** Potential succinylated drugs and their impact on cancer.

Compounds	Target Protein	Corresponding Cancer	Succinylated Protein	Impacts on Cancer	Year	Ref.
Aspirin	NF-κB p65/HAT1	Hepatocellular carcinoma	PGAM1 K99suc (reduced)	Inhibition of cancer progression	2023	[111]
AS-IV	KAT2A	Hepatocellular carcinoma	PGAM1 Ksuc (reduced)	Inhibition of cancer progression	2024	[26]
Lidocaine	SIRT5	Lung adenocarcinoma	HIST1H2BL Ksuc (reduced)	Inhibition of cancer progression	2024	[113]
Glibenclamide	CPT1A	Ovarian cancer	MFF Ksuc (reduced)	Inhibition of cancer progression	2025	[91]
Catalpol		Breast cancer	Tumor protein Ksuc(reduced)	Inhibition of cancer progression	2022	[117]

## Abbreviations

ACOX2	branched-chain acyl-CoA oxidase
AKT/mTOR	protein kinase B/mammalian target of rapamycin
α-KDGHC	α-ketoglutarate dehydrogenase complex
α-KG	α ketoglutaric acid
AS-IV	astragaloside IV
ATC	anaplastic thyroid cancer
BC	bile acid
CA	cholic acid
COX-2	cyclooxygenase-2
CPT1A	carnitine palmitoyltransferase 1A
CS	citrate synthase
CTBP1	C-terminal binding protein 1
EIF3B	eukaryotic translation initiation factor 3 subunit B
EMP	Embden–Meyerhof–Parnas pathway (glycolysis)
FBN1	fibrillin 1
GPI	glucose-6-phosphate isomerase
HAT1	histone acetyltransferase 1
IDH2	isocitrate dehydrogenase 2
KAT2A	lysine acetyltransferase 2A
KIF23	kinesin family member 23
Ksuc	succinyl-lysine
LACTB	β-lactamase-like protein
LDHA	lactate dehydrogenase A
LRPPRC	leucine-rich PPR motif-containing protein
MCM	methylmalonyl-CoA mutase
ME2	mitochondrial malic enzyme 2
MFF	mitochondrial fission factor
NPM1	nucleophosmin 1
OXCT1	3-oxoacid CoA-transferase 1
P4HA1	proline 4-hydroxylase α subunit
PDHA1	pyruvate dehydrogenase E1 subunit α1
PDPK1	phosphoinositide-dependent protein kinase-1
PGAM1	phosphoglycerate mutase 1
PGK1	glycolytic enzyme phosphoglycerate kinase 1
PKM2	pyruvate kinase M2
PRMT5	protein arginine methyltransferase 5
PTC	papillary thyroid cancer
SIRT5	sirtuin 5
SP5	specificity protein 5
SUCLA2	succinate-CoA ligase ADP-forming subunit β2
SUCLG2	succinate-CoA ligase GDP-forming subunit β2
TAGLN2	transgelin 2
